# Editorial: Genetic and epigenetic regulation of disease resistance in horticultural plants

**DOI:** 10.3389/fgene.2023.1277571

**Published:** 2023-09-04

**Authors:** Muhammad Amjad Ali, Feng Zhang, Jiantao Guan, Yue Ma

**Affiliations:** ^1^ Department of Plant Pathology, University of Agriculture, Faisalabad, Faisalabad, Pakistan; ^2^ Chinese Academy of Agricultural Sciences (CAAS), Beijing, China; ^3^ College of Horticulture, Shenyang Agricultural University, Shenyang, China

**Keywords:** horticultural crops, genetic regulation, disease resistance, genome editing, host induced gene silencing (HIGS)

## Introduction

Horticultural plants include a wide variety of plants that are important for supplying food and fuel as well as improving the aesthetic appeal of many habitats and ecosystems (Okunlola et al., 2016). These plants are primarily grown in solar greenhouses and plastic greenhouses, where they are susceptible to disease, endure wide temperature swings, and have high humidity levels (Arshad et al., 2022). Unfortunately, disease outbreaks and an overuse of pesticides are impeding the expansion of the horticultural business. Effective disease prevention techniques must address these issues by choosing germplasm that is resistant to disease and creating resilient plant varieties through breeding and genetic modification (Zahoor et al., 2020; Shaw et al., 2021).

The discovery of novel candidate genes and research into the molecular processes behind disease resistance in horticultural plants are crucial steps in this regard. Many horticultural plant species now have high-quality, full reference genomes available thanks to advances in third generation sequencing techniques, enabling integrated analysis of multi-omics data using bioinformatics tools (Debener, 2022; Jabran et al., 2023). These analyses help us better understand the evolution of horticultural plants and provide useful genomics resources for research on disease resistance features in plants. We can significantly change the genetic makeup of agricultural plants to increase their disease resistance by gaining knowledge of their genetics and epigenetics (Xanthopoulou et al., 2023).

This research area examines the genetic and epigenetic regulation of disease resistance in horticultural plants considering the aforementioned findings. Along with well-established genes, this area also sought to find novel genes and understand their activities and regulatory mechanisms, offering a variety of possibilities for improving the genetic potential of horticultural plants. The development of quantitative trait loci (QTL) mapping and genome-wide association studies (GWAS) were the key themes in this research, which sought to understand the genetic basis of disease resistance in horticultural plants. The second theme is the investigation of epigenetic mechanisms, such as DNA, RNA, and Histone alteration, that control disease resistance in horticultural plants. Additionally, the goal of this topic was to compile information on recent developments in the use of genetic and epigenetic strategies to improve disease resistance in horticultural plants as well as the use of multi-omics data sets that include information on the genome, transcriptome, proteome, metabolome, epigenome, and/or microbiome to better understand disease resistance in these plants.

## Insights from the Research Topic

In this Research Topic several research and review articles have been published that have deepened our knowledge about the recent advancements in genetic and epigenetic regulation of disease resistance in horticultural plants. For instance, dieback poses a serious threat to *Dalbergia sissoo*, a crucial tree in forestry, agroforestry, and horticulture. Numerous outbreaks have killed billions of *D. sissoo* trees, necessitating immediate effort to identify the cause of the dieback. In this Research Topic, Haq et al. used phylogenomics to link Ceratocystis species to *D. sissoo* mortality. Through morphological analysis, fungi isolated from tissues of the plants suffering from dieback were distinguished from Fusarium wilt. They concluded that the *Ceratocystis fimbriata sensu lato* complex is the cause of Pakistan’s shisham dieback. Additional genomic and phylogenetic analysis identified *Ceratocystis dalbergicans* sp. *nov*. as the unique species responsible for the dieback disease in *D. sissoo* in Pakistan. This extraordinary finding provides an essential starting point for understanding and minimizing the potential of dieback in this commercially important tree species. The NBS-LRR gene family, which is linked to plant disease resistance, was examined by the same research team in *D. sissoo* (Ijaz et al.). They examined the transcriptome using DOP-rtPCR and discovered distinctive motifs that suggested the presence of genes for disease resistance. They added that their research provides helpful insights into potential genetic factors that may affect dieback resistance in this significant species of timber tree.

Citrus cultivars are at serious risk from citrus viroid infection because it spreads swiftly through infected pruning tools and quickly goes throughout the host plant’s system. In order to better comprehend and address this issue, Ali et al. investigated the distribution and molecular features of Citrus bent leaf viroid (CBLVd) and its variations in numerous citrus varieties. CBLVd infection was present in 154 samples of symptomatic citrus at a rate of 36.33%, according to the research. Their findings revealed that the shisham dieback in Pakistan is attributed to the *C. fimbriata sensu lato* complex. Through further genomic and phylogenetic investigation, *C. dalbergicans* sp. *nov*. was identified as the specific causative agent behind the dieback disease in *D. sissoo*. This remarkable discovery serves as a crucial initial step towards comprehending and mitigating the risk of dieback in this economically important tree species. The research team also explored the NBS-LRR gene family, associated with plant disease resistance, in *D. sissoo* (Ijaz et al.). Employing DOP-rtPCR to analyze the transcriptome, they detected distinct motifs indicating the presence of disease resistance genes. Moreover, they highlighted that their study offers valuable insights into potential genetic factors influencing dieback resistance in this significant timber tree species.

The vulnerability of citrus cultivars to citrus viroid infection is a serious concern due to its rapid transmission via contaminated pruning equipment and its swift dissemination within the host plant. To enhance our understanding and devise effective solutions, Ali et al. conducted a study to examine the dispersion and molecular characteristics of Citrus bent leaf viroid (CBLVd) and its different forms across a range of citrus cultivars. CBLVd infection was present in 154 samples of symptomatic citrus at a rate of 36.33%, according to the research. They used biological indexing on Etrog citron to examine the effects of CBLVd on citrus trees, noting smaller leaves, a yellowing with a light green pattern, and bending. Ali et al. sequenced and evaluated the amplified products in our molecular investigation, which showed a remarkable 98% similarity with other CBLVd isolates. Additionally, two main groups (A and B) were revealed by the phylogenetic tree analysis, with the CBLVd CVd-I-LSS sequences primarily clustered in subgroup A1. It is interesting to note that we also discovered a new host of CVd-I-LSS in Pakistan: Palestinian sweet lime. Notably, the diversity of the nucleotide sequence rendered CBLVd undetectable by standard primers, highlighting the need for new detection techniques. These results highlight the criticality of putting in place efficient management and quarantine measures to stop the spread of CBLVd and protect citrus crops in Pakistan and other impacted areas.

*Fusarium oxysporum* f. sp. *phaseoli* (Fop) is the culprit behind the widespread disease known as fusarium wilt in common beans. The management of diseases may benefit from genes involved in the Fop response. In this Research Topic, Liu G. et al. looked into the crucial regulator of plant pathogen defense, the TGA-binding transcription factor (*TGA*). They identified and characterized eight *TGA* genes in common beans using thorough genome analysis, which were divided into four subgroups with different sequence architectures. During Fop inoculation and salicylic acid treatment, *PvTGA03* and *PvTGA07* showed notable variations in expression levels between resistant and susceptible genotypes. These discoveries increase our understanding of the *PvTGA* gene family and might improve common beans’ resistance to Fusarium wilt. Another research group working on common beans reported the newly conducted research on the accumulation of bean common mosaic virus in *Phaseolus vulgaris* demonstrates disease resistance during the early phase of infection (Çelik et al.). They developed a unique quantitative real-time PCR (qRT-PCR) technique based on SYBR Green that targets the coat protein gene. Through melting curve analysis, the method showed great specificity and validity, ensuring no cross-reactions. They evaluated twenty different common bean genotypes, and the results showed variable degrees of sensitivity to the BCMV strain, with the most resistant and vulnerable genotypes being designated as YLV-14 and BRS-22, respectively. The accurate BCMV quantification made possible by the qRT-PCR provided useful information for the early selection of resistant genotypes in bean tissues (Çelik et al.).

Similarly, disease outbreaks and overuse of pesticides pose serious problems for the horticultural sector. MicroRNAs, crucial elements of eukaryotic transcriptomes, control the expression of genes at all stages of plant development. Zhang et al. reviewed the recent developments in short RNA sequencing that have made it possible to study how horticultural plants regulate their genes for defense against invading pathogens. They summarize the state of knowledge on microRNA biogenesis and their roles in disease resistance in horticultural plants in this review. These microRNAs have the potential to be extremely useful genetic resources for improving disease resistance and developing molecular breeding techniques. The paper also discusses the difficulties in understanding the biology of horticultural plant microRNAs and considers the possibility of using these gene resources to enhance disease resistance in the future.

According to Liu Y. et al., tomato reproduction is significantly hampered by *Cladosporium fulvum* invasion. They comprehend the defense response mechanism of a *Cf-10* gene-carrying line, which demonstrated notable resistance to *C. fulvum* and discovered 54 differently expressed miRNAs (DE-miRNAs) and 3,016 differentially expressed genes (DEGs) in the *Cf-10* gene-carrying line by multiple-omics profiling at non-inoculation and 3 days post-inoculation stages. These DE-miRNAs may have influenced hormone signaling pathways at the interface of interactions between plants and pathogens, which would have impacted immunity. A crucial regulator of SA synthesis, *SARD1*, may have been increased by *miR9472* downregulation leading to an increase in SA levels. These findings expand our knowledge of tomato resistance mechanisms against *C. fulvum* by revealing a thorough genetic circuit and useful gene targets for improving resistance to this disease in the Cf-10-carrying line.

## Future perspectives

Several future directions hold promise for enhancing agriculture and food security by addressing the problems caused by significant diseases affecting horticultural crops and halting the spread of dangerous pathogens. While producing various disease-resistant types is critical for sustainable agriculture, which benefits both farmers and the environment, disease-resistant cultivars are still important. Remote sensing technologies can improve disease surveys by supplying more thorough and trustworthy data. The advancement of horticultural crops research can be accelerated, and worldwide competitiveness can be improved, by integrating molecular breeding efforts among national program partners. Genomic transformation is still a useful tool for figuring out how genes work and discovering novel sequences. The identification of pathogen-derived resistance mechanisms, such as posttranscriptional gene silencing (PTGS), creates prospects for PTGS-based resistance characteristics, which are frequently strong (Ali et al., 2017).

Host-induced gene silencing (HIGS) and peptide-based approaches are vital for targeted gene control and provide plants with defense against a variety of diseases. Modern methods like virus-induced gene silencing (VIGS), HIGS, and mutant screening have been widely used in the search for new resistance genes (Sawyer and Labbé, 2021). The opportunity for broad spectrum sequence modifications to improve disease resistance in plant genes is provided by sequence-specific nucleases (SSNs).

Advanced biotechnologies and stringent plant quarantine regulations are essential for stopping infections at their source. The ability to identify unknown infections using new plant biotechnologies helps with quarantine regulations and enables early action. Looking ahead, embracing novel biotechnologies will considerably assist concentrating on the molecular breeding of highly attractive horticultural crop cultivars. We can improve the diagnosis and management of horticultural crop infections by bringing together cutting-edge techniques, ongoing research, and stakeholder participation. This will guarantee a sustainable and resilient production system for horticultural crops to satisfy the expanding demands of a global population.
